# Comparison of the Structure and Properties of Hydroxypropyl Starch/Carrageenan Blends with Different Amylose/Amylopectin Contents

**DOI:** 10.3390/gels12050423

**Published:** 2026-05-12

**Authors:** Xingxing Zhu, Di Wu, Juanjuan Wu, Jinglong Zhao, Yunhe Lian, Yunkai Lv

**Affiliations:** 1 College of Chemistry and Materials Science, Hebei University, Baoding 071002, China; zhuxingxing0089@126.com (X.Z.);; 2 Chenguang Biotechnology Group Co., Ltd., Handan 057250, China; 3 Chenguang Biotechnology Group Handan Co., Ltd., Handan 056007, China; 4 Hebei Nutrition and Health Food Technology Innovation Center, Handan 056004, China

**Keywords:** polysaccharide complex, carrageenan, amylose, amylopectin, interaction mechanism

## Abstract

To compare the structure and properties of hydroxypropyl starch/carrageenan blends with different amylose/amylopectin contents, two types of hydroxypropyl starch—a high-amylose type (amylose content > 70%) and a high-amylopectin type (amylopectin content > 95%)—were used. These starches had similar molecular weights, degrees of hydroxypropyl substitution, and other properties, differing only in their amylose and amylopectin contents. Each starch was blended with carrageenan via a solution blending method, and the resulting blends were systematically characterized by Fourier transform infrared spectroscopy (FTIR), X-ray diffraction (XRD), thermogravimetric analysis, rheological tests, texture analysis, mechanical property tests, contact angle analysis, and UV-Vis spectrophotometry. The results showed that, upon blending with carrageenan, the hydroxypropyl starch transformed from a weak viscoelastic solution into an elastic, strong gel. FTIR and XRD analyses confirmed that the hydroxypropyl starch and carrageenan formed a homogeneous, compact, three-dimensional network via hydrogen bonding. This significantly enhanced the mechanical strength and stability of the blends. The influence of starch molecular structure on the blend system’s properties exhibited a pronounced state dependence. In the gel state, hydroxypropyl amylopectin effectively filled the carrageenan network due to its high swelling capacity, thereby improving the thermal stability and textural properties of the blends. However, in the film state, hydroxypropyl amylose with higher crystallinity and denser molecular packing contributed to superior tensile strength, hydrophobicity and light transmittance. Furthermore, the optimal mass ratio of hydroxypropyl starch to carrageenan was found to be in the range of 2:1 to 4:1. With this ratio, excessive cross-linking and poor compatibility could be avoided, resulting in improved mechanical performance, hydrophobicity, and light transmittance. This study reveals the relationship between starch molecular structure, system state and macroscopic properties, providing a theoretical basis for the rational design and regulation of the properties of hydroxypropyl starch/carrageenan blends.

## 1. Introduction

Starch is a polysaccharide consisting mainly of the two glycosidic polymers amylose and amylopectin. It exists in the form of porous, semi-crystalline granules and exhibits strong hydrophilicity. Due to its wide availability, low cost and inherent biodegradability, starch has become an important raw material for the development of functional materials [1]. However, limitations of native starch include low gel strength, high retrogradation tendency, and inadequate thermal stability, which restrict its direct application. To address these limitations, etherification modification is commonly employed to prepare hydroxypropyl starch, thereby improving its hydrophilicity, film-forming ability, and processability [2]. Given the strong hydrophilicity of starch, it is often blended with non-starch polysaccharides, such as hydrophilic colloids, for practical applications. This strategy protects starch granules during processing, improves the texture and rheological properties of the system, and imparts excellent water retention and water barrier performance. Carrageenan, a typical marine-derived natural polysaccharide, is often compounded with starch to create a blend with superior gel properties, leveraging its exceptional gelling ability [3]. Due to the availability of raw materials, the maturity of processing technology, and the tunability of properties, this blending system shows promise in fields such as food, pharmaceuticals, and packaging materials [4].

In recent years, significant progress has been made with the hydroxypropyl starch/carrageenan blend across various fields. In the area of edible films and packaging materials, studies have shown that this blend can form a dense and uniform film structure through hydrogen bonding, effectively enhancing the mechanical strength, water solubility, and barrier properties of single-polysaccharide films [5]. By further incorporating functional components such as nanocellulose or gum ghatti, rapidly water-soluble, green, and biodegradable edible films have been successfully prepared and widely applied in solid beverage packaging [6] and fruit and vegetable preservation [7]. Furthermore, smart label technology based on pea starch and carrageenan loaded with plant extracts has enabled visual monitoring of the freshness of fresh produce [8]. In the field of food processing, the combination of carrageenan with starch and protein has been shown to significantly improve textural properties. For example, adding carrageenan and sweet potato protein hydrolysate to starch-based fresh wet noodles was found to enhance tensile strength while reducing the glycemic index [9]; incorporating carrageenan into meat-free sausages effectively reduced cooking loss and improved mouthfeel [10]; in surimi, meat products, and similar systems, this blend enhanced gel strength and water-holding capacity, improving freeze–thaw stability and storage quality [11]. In the realm of drug delivery carriers, the hydroxypropyl starch/carrageenan blend not only inhibits starch retrogradation but also increases material toughness, significantly improving the mechanical properties and stability of capsule shells, thereby providing a feasible technological pathway for the development of plant-based soft capsules [12].

Although significant advances have been made in optimizing the properties of starch/carrageenan blends, most studies have focused on the macroscopic blending and performance enhancement of native starch or conventionally modified starch with carrageenan. Systematic comparative investigations on the blending characteristics of starches with varying amylose and amylopectin contents remain limited. In fact, amylose and amylopectin exhibit marked differences in molecular structure, and their ratio is a key factor influencing starch gelatinization, gelation, and recrystallization behavior [13]. Amylose readily forms double helical structures and tends to recrystallize into firm gels, whereas amylopectin, owing to its highly branched molecular architecture, forms gels with a more fragile texture [14]. These differences consequently affect the properties of the system after compounding with carrageenan.

Based on this, the present study employs hydroxypropyl amylose and hydroxypropyl amylopectin as the core raw materials to systematically investigate the distinct effects of these two starch components on the rheological behavior, gel texture, mechanical properties, surface characteristics, and optical properties of starch/carrageenan blends. By elucidating the intrinsic relationship between starch molecular structure and the properties of the blend, this study aims to provide a fundamental theoretical basis for the property regulation of starch/carrageenan blends.

## 2. Results and Discussion

### 2.1. Analysis of Basic Physicochemical Properties of Hydroxypropyl Starch and Carrageenan

The basic physicochemical properties of the two hydroxypropyl starches, which differ in molecular structure, and of carrageenan used in the experiment are presented in Table 1 and Table 2, respectively. Both starches were hydroxypropyl-modified. Specifically, hydroxypropyl high-amylose starch (amylose content 70%) and hydroxypropyl high-amylopectin starch (amylopectin content 95%), hereinafter referred to as hydroxypropyl amylose (HS) and hydroxypropyl amylopectin (HP), respectively. These two starches exhibited similar hydroxypropyl contents (4.33% and 4.30%), with a pH of 6.2, indicating weak acidity and essentially consistent properties. However, a significant difference in viscosity was observed between the two starches: the viscosity of HS was 20.10 mPa·s, whereas that of HP was 396.00 mPa·s. Both starches displayed low solubility at room temperature. As the temperature increased, the solubility gradually improved and the swelling capacity correspondingly increased. In addition, owing to its abundant short-chain structure, HP exhibited a higher swelling capacity than HS.

The carrageenan used in the experiment was type ι–carrageenan (IC), which exhibited good gel strength and stable, uniform physicochemical properties at a mass fraction of 1.5%, meeting the requirements for subsequent experiments.

**Figure 1:**
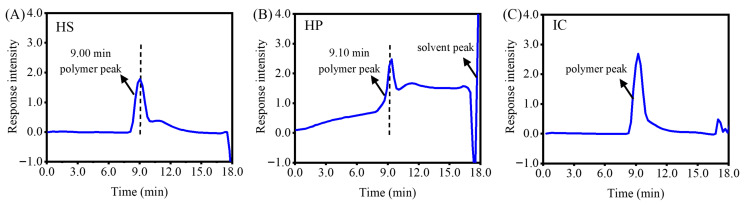
Molecular weight distribution curves of hydroxypropyl amylose (HS, (**A**)), hydroxypropyl amylopectin (HP, (**B**)), and ι-carrageenan (IC, (**C**)).

Gel permeation chromatography is based on the molecular sieving effect of porous gels and separates analytes according to their hydrodynamic volume, enabling the determination of polymer molecular weight distribution. The ordinate represents the refractive index difference between the sample and the solvent, with the response signal of the differential refractive index detector being proportional to the solute concentration; the abscissa represents the retention time. According to the principle of gel permeation chromatography, compounds elute in order of decreasing molecular weight. As shown in Figure 1, the peak at 9 min corresponds to the main polymer peak, while the peaks at 18 min and beyond are attributed to the elution solvent. By comparing the peak intensities and peak positions, it can be observed that the molecular weights and distributions of the two starches are similar (Figure 1).

In summary, to investigate the effect of starch structure on properties, this study selected two hydroxypropyl starches that differed only in amylose and amylopectin contents, while keeping other physicochemical properties (such as hydroxypropyl content, pH, molecular weight, etc.) consistent. This design eliminated the interference of non-structural factors, thereby establishing an experimental foundation for accurately elucidating the influence of starch molecular structure on the properties of the blend.

### 2.2. Rheological Properties of Hydroxypropyl Starch/Carrageenan Blends

#### 2.2.1. Strain Sweep Analysis

**Figure 2:**
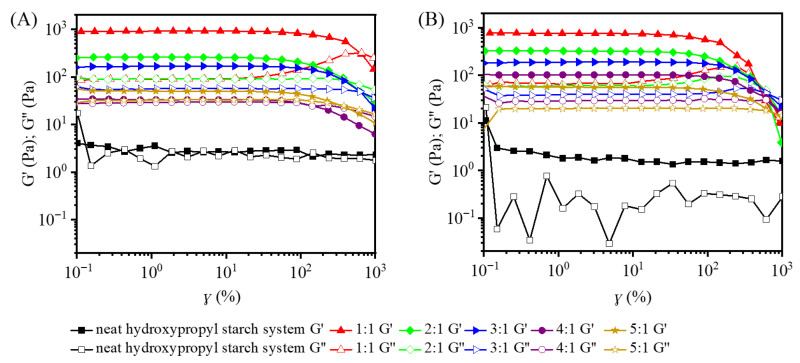
Strain-dependent relationship between G′ and G″ for hydroxypropyl amylose (HS)/carrageenan blends (HSC, (**A**)) and hydroxypropyl amylopectin (HP)/carrageenan blends (HPC, (**B**)).

In dynamic rheology, the storage modulus (G′) and loss modulus (G″) serve as two fundamental descriptors of viscoelastic behavior. G′ captures the elastic component—that is, the material’s capacity to store deformation energy—while G″ captures the viscous component, reflecting the energy dissipated during deformation [15]. To probe the structural integrity of the hydroxypropyl starch/carrageenan blends, the dependence of both moduli on applied strain was examined, and the results are presented in Figure 2. Over the 0.1–1000% strain range, the neat HS system alone showed only minor fluctuations in G′ and G″, with both moduli hovering around comparable values (2.46 Pa ± 0.51 Pa). Under the same deformation range, the neat HP system likewise maintained a relatively stable G′ (1.78 Pa ± 0.43 Pa); however, G″ remained consistently below G′ and exhibited much larger scatter (0.03–0.76 Pa) because its magnitude was very low (Figure 2B), indicating that both neat hydroxypropyl starch systems exhibited weak viscoelastic characteristics.

The strain range over which the G′ and G″ of a material remain constant is defined as the linear viscoelastic region (LVR). Within this region, the internal network structure of the gel remains intact and deformation is reversible. Strain sweep results showed that after compounding hydroxypropyl starch with carrageenan, the LVR of the system ranged from 0.1% to 100%. When the strain exceeded the LVR, G′ gradually decreased with increasing strain, while G″ first increased and then decreased, indicating that the weak intermolecular interactions were progressively disrupted, ultimately leading to the collapse of the gel network structure. When the proportion of carrageenan was low, the blend exhibited a low modulus, and the gel properties were primarily governed by the starch component. As the proportion of carrageenan in the blends increased, both G′ and G″ increased significantly, attributed to the enhanced hydrogen bonding interactions between carrageenan and starch molecules with increasing carrageenan concentration, thereby improving the elasticity of the gel network. For example, at a strain of 1%, when the hydroxypropyl starch-to-carrageenan blending ratio reached 1:1, G′ increased from 2.27 Pa at the 5:1 ratio to 872.54 Pa, and G″ increased from 1.19 Pa to 78.76 Pa, with G′ exceeding G″ by approximately one order of magnitude, indicating that the system exhibited typical solid-like behavior with maximum elasticity and intermolecular interactions.

A further comparison of the two starch types revealed that at a hydroxypropyl starch-to-carrageenan ratio of 5:1, the HSC blend already exhibited elastic dominance (G′ > G″), but the moduli were close in value and the elastic advantage was weak (Figure 2A). At the same ratio, the HPC blend also showed elastic dominance, yet the gap between G′ and G″ was larger (although not yet an order of magnitude), indicating a more pronounced elastic character (Figure 2B).

#### 2.2.2. Frequency Sweep Analysis

**Figure 3:**
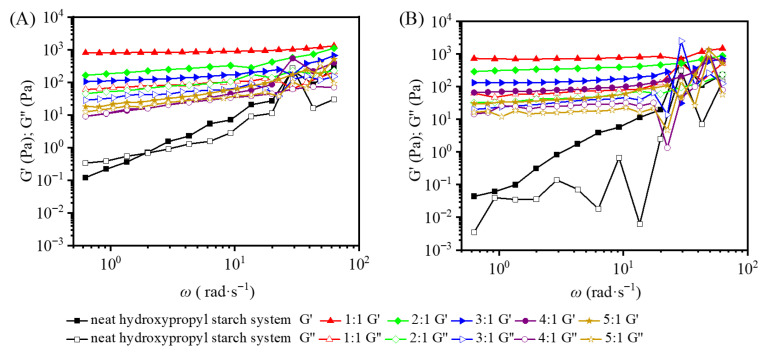
Relationship between G′, G″ and angular frequency for hydroxypropyl amylose (HS)/carrageenan blends (HSC, (**A**)) and hydroxypropyl amylopectin (HP)/carrageenan blends (HPC, (**B**)) at a constant strain of 1%.

Frequency sweep tests provide a useful window into the viscoelastic character of blends and their frequency dependence. All measurements were carried out at a fixed strain of 1%, and the resulting modulus–angular frequency curves for the various starch/carrageenan ratios are shown in Figure 3. The loss factor tan δ (defined as G″/G′) is commonly used to distinguish solid-like from liquid-like behavior: when tan δ < 1 (G′ > G″), elastic network contributions dominate and the material responds in a solid-like manner; when tan δ > 1 (G″ > G′), viscous flow prevails and the system behaves more like a liquid [16]. Ahmad et al. [17] pointed out that the starch gel system can be regarded as a three-dimensional network structure formed by swollen starch granules as the dispersed phase embedded in a continuous phase dominated by amylose. Both the neat HS and neat HP systems exhibited only weak viscoelasticity. Across the full angular frequency range tested (0.628–62.800 rad·s^−1^), G′ and G″ were close in value and each varied by more than 20-fold with frequency, reflecting a strong frequency dependence and the absence of a well-developed gel network.

After the addition of carrageenan, the sulfate groups on its molecules interact with amylose in the starch gel system through hydrogen bonding and electrostatic interactions. Meanwhile, during heating, carrageenan forms double helices that aggregate with each other, thereby increasing the number of physical crosslinking points in the blend and rendering the original network structure more compact and continuous, ultimately constructing an elasticity-dominated three-dimensional crosslinked network [18]. Frequency sweep results indicated that the incorporation of carrageenan significantly improved the viscoelasticity and structural stability of the system, forming a stronger elastic gel compared with the neat starch system.

Polysaccharide gels can be classified into strong gels (tan*δ* < 0.1) and weak gels (tan*δ* > 0.1) based on the loss tangent (tan*δ*). For the HPC blends (Figure 3B), G′ remained above G″ throughout the low to medium-high frequency range of 0.1–10 rad·s^−1^, indicating that elastic response dominated. Under the high carrageenan condition with a hydroxypropyl starch-to-carrageenan ratio of 1:1, the frequency dependence of G′ and G″ was significantly weakened, and the moduli remained nearly constant with changes in angular frequency. At a fixed angular frequency of 6.28 rad·s^−1^, the blend had a G′ value of 749.84 and a tanδ value of 0.10, exhibiting typical rheological characteristics of a strong gel [19]. Similarly to the HPC blends, carrageenan also enhanced the stability of the HSC (Figure 3A). However, these blends exhibited lower moduli at low frequencies and stronger frequency dependence, indicating slightly lower gel stability compared with the HPC blends. In the frequency range of 0.1–10 rad·s^−1^, at a blending ratio of 1:1, the fluctuations of both moduli with angular frequency were relatively weak. At a fixed angular frequency of 6.28 rad·s^−1^, the blend showed a G′ value of 864.66 and a tan*δ* value of 0.12. The slightly higher G′ and tan*δ* values indicate that the HSC blend forms a denser elastic network but with less uniform cross-linking and weaker structural stability.

Meanwhile, for the neat hydroxypropyl starch system and the blends with low carrageenan content, when the angular frequency exceeded 10 rad·s^−1^, the fluctuations in G′ and G″ became significantly larger and irregular. A comparison revealed that the HSC blends showed smaller modulus variations in the high-frequency region than the HPC blends. It is speculated that this is because the linear molecular structure of HS forms a more regular network with carrageenan featuring more robust crosslinking points. Additionally, the higher amylose content facilitates the reinforcement of crystalline structures through molecular chain rearrangement, making the blend less prone to localized dissociation under high-frequency shear and thus conferring stronger resistance to shear-induced disruption.

#### 2.2.3. Temperature Sweep Analysis

**Figure 4:**
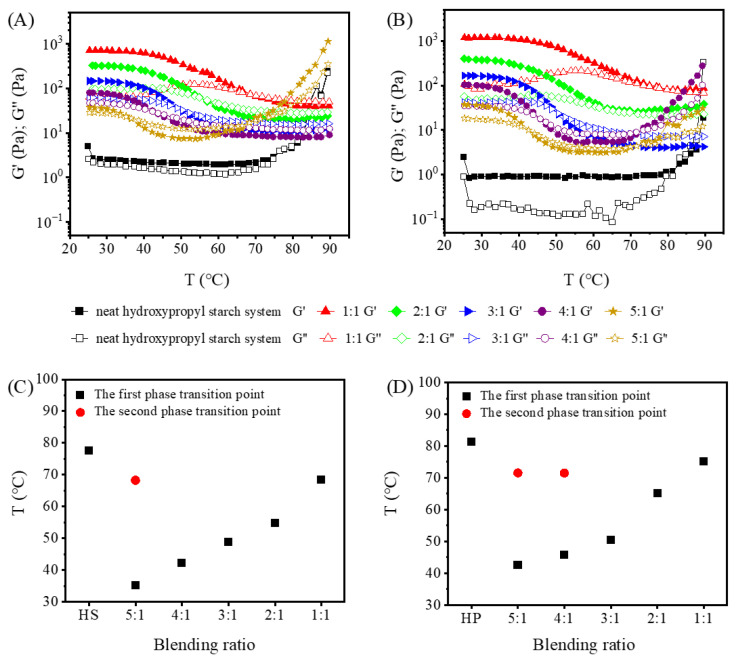
The temperature-dependent curves of G′ and G″ for the blend systems of hydroxypropyl amylose (HS)/carrageenan (HSC, (**A**)) and hydroxypropyl amylopectin (HP)/carrageenan (HPC, (**B**)), along with the corresponding crossover temperature (G′ = G″) plots for the two blends (**C**,**D**).

Temperature-dependent rheological measurements were performed to elucidate the thermally induced phase transitions of the hydroxypropyl starch/carrageenan blends and to determine the onset temperature for elastic network formation as well as the overall thermal stability. The evolution of G′ and G″ with increasing temperature for blends at different hydroxypropyl starch-to-carrageenan ratios is shown in Figure 4.

For the neat HS system (Figure 4A), the moduli remained nearly constant at 1.81 ± 0.31 Pa as the temperature climbed toward 70 °C, after which they rose sharply and simultaneously, reaching 236.28 Pa by 90 °C. Throughout the entire heating ramp, G′ and G″ stayed close in magnitude. The neat HP system (Figure 4B) behaved differently during heating: G″ fluctuated slightly, while G′ consistently exceeded G″ by roughly one order of magnitude, until around 80 °C when a simultaneous surge occurred and the system began to transition from an elastically dominated state to a viscous state. The heating-induced increase in the moduli of the neat hydroxypropyl starch systems is primarily attributed to starch granule swelling, amylose leaching, and the concomitant expansion of the gel phase. Once carrageenan was incorporated, all blends exhibited a broadly similar thermal profile regardless of starch type: the moduli first decreased progressively with rising temperature and then leveled off. This trend is ascribed to the progressively enhanced molecular motion at elevated temperatures, which enlarges intermolecular distances, weakens intermolecular interactions, relaxes the gel network, and lowers the viscosity—consequently reducing the moduli [20].

During the temperature sweep, the intersection point of G′ and G″ shifted to progressively higher temperatures with increasing carrageenan content in both the HPC and HSC blends. This was because as the carrageenan content increased, its polysaccharide chains inserted into the double helical structure of starch molecules, enhancing intermolecular crosslinking and making the spatial network structure of the gel more compact. Consequently, a higher temperature was required to disrupt its rigid structure. Moreover, at the same blending ratio, the intersection temperature (gel point) of the HPC blend was approximately 2–10 °C higher than that of the HSC blend, indicating that the HSC blends could undergo gelation at a lower temperature and thus more readily form a gel structure. In contrast, the gel network formed by the HPC blends maintained stronger structural integrity at higher temperatures, requiring more energy for network disintegration, and therefore exhibited superior temperature resistance.

An intriguing phenomenon was also noted in this study. Specifically, the HSC blend at a hydroxypropyl starch-to-carrageenan ratio of 5:1, and the HPC blend at ratios of 5:1 and 4:1, all displayed two modulus crossovers during heating: G′ > G″ between 25 and 40 °C, G′ < G″ between 40 and 70 °C, and G′ > G″ again between 70 and 90 °C. A tentative interpretation is as follows. At low temperatures (25–40 °C), the double-helical aggregates of carrageenan provide the primary elastic framework, with starch chains serving as auxiliary reinforcement. In the intermediate range (40–70 °C), the carrageenan network begins to melt, G′ decreases, and the blend temporarily becomes viscosity-dominated. At higher temperatures (70–90 °C), the starch chains and the now-disordered carrageenan molecules re-cooperate, restoring elastic dominance. This behavior was observed only in blends with relatively low carrageenan content; when the carrageenan concentration is high enough, the carrageenan chains self-associate at elevated temperatures to form a dominant network, and the second crossover disappears.

### 2.3. FTIR Analysis of Hydroxypropyl Starch/Carrageenan Blends

**Figure 5:**
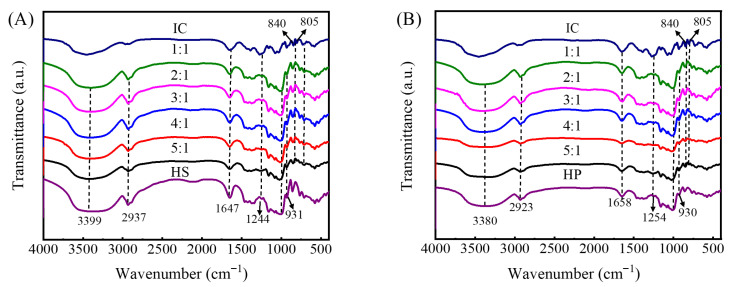
Infrared spectra of hydroxypropyl amylose (HS)/carrageenan (IC) blends (HSC, (**A**)) and hydroxypropyl amylopectin (HP)/carrageenan (IC) blends (HPC, (**B**)).

FTIR spectra of hydroxypropyl starch/carrageenan blends prepared at varying ratios are presented in Figure 5. The spectrum of the neat hydroxypropyl starch system exhibited absorption bands typical of carbohydrates: an O–H stretching band reflecting intermolecular hydrogen bonding (3200–3400 cm^−1^), C–H stretching (2920–2930 cm^−1^), the H–O–H bending vibration of adsorbed water (1640–1650 cm^−1^) [21], and the polysaccharide backbone fingerprint displayed with C–O–C and C–O stretching modes (900–1200 cm^−1^) [22]. A comparison of the two starch types revealed that amylose gave rise to sharper and more prominent peaks in the 3200–3400 cm^−1^ and 1643 cm^−1^ regions (Figure 5A), whereas the corresponding absorptions of amylopectin were broader and less defined (Figure 5B), reflecting differences in molecular chain regularity. The characteristic absorptions of carrageenan were concentrated in the sulfate ester and sugar ring vibration regions: the band at 1220–1260 cm^−1^ was assigned to the asymmetric S=O stretching of sulfate ester groups [23]; absorptions at 840 and 805 cm^−1^ indicated the presence of sulfate substituents at the C4 and C2 positions, respectively [24].

The hydroxypropyl starch/carrageenan blends retained the main characteristic peaks of both polysaccharides, and no new absorption bands appeared, confirming that no chemical reaction took place and that the interactions between hydroxypropyl starch and carrageenan are predominantly physical in nature. The structural integrity of each component’s functional groups preserved [25]. As the proportion of carrageenan increased, the O–H stretching band near 3400 cm^−1^ gradually intensified and narrowed slightly, indicating more ordered and uniform intermolecular hydrogen bonding networks between the two polysaccharides [26]. In the fingerprint region, the S=O stretching band of sulfate esters (1220–1260 cm^−1^) and the characteristic peaks related to 3,6-anhydrogalactose and D-galactose-2-sulfate (at 930 and 805 cm^−1^) intensified with increasing carrageenan proportion, confirming that the carrageenan structure remained stable in the blends and that its content variation could be accurately reflected by the intensity of these bands.

### 2.4. XRD Analysis of Hydroxypropyl Starch/Carrageenan Blends

**Figure 6:**
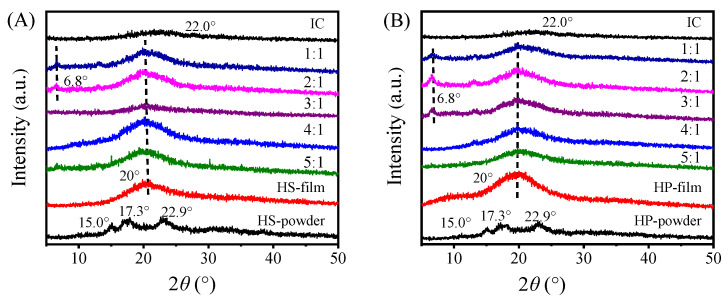
XRD patterns of hydroxypropyl amylose (HS)/carrageenan (IC) blends (HSC, (**A**)) and hydroxypropyl amylopectin (HP)/carrageenan (IC) blends (HPC, (**B**)).

XRD patterns of hydroxypropyl starch/carrageenan blends at different ratios are shown in Figure 6. Ungelatinized starch exhibited diffraction peaks at 15°, 17.3°, and 22.9°, whereas gelatinized starch showed only a neat broad peak near 20°, which is characteristic of an amorphous structure. This confirms that the long-range crystalline order of the starch granule was disrupted during gelatinisation, leaving the system predominantly composed of amorphous chain segments with limited short-range order.

Following blending with carrageenan, all formulations retained the broad diffraction peak around 20°. Compared with neat hydroxypropyl starch and carrageenan films, no new diffraction peaks were observed, indicating good compatibility between the two components and predominantly physical interactions without chemical reactions. As the proportion of hydroxypropyl starch decreased, the peak position remained unchanged while its relative intensity gradually weakened. This indicates that crystallinity diminishes with declining starch content, and that the diffraction signal is primarily contributed by the starch component. In blends with low carrageenan content, hydrogen bonds and van der Waals forces between the starch molecules dominate, resulting in higher crystallinity and sharper, more intense peaks. At high carrageenan loadings, however, the carrageenan chains occupy a substantial volume fraction, disrupting the orderly packing of starch molecules and suppressing crystalline domain formation. This leaves the system largely amorphous and accounts for the weaker diffraction signal [27].

An additional weak peak at 6.8° was also detected in blends with high proportions of carrageenan. According to Janaswamy and Chandrasekaran [28] the interhelical packing distance of ι-carrageenan ranges from 12.2 Å to 13.9 Å, corresponding to a Bragg diffraction angle of approximately 6–7°, which is consistent with the observations in this study. Therefore, the diffraction peak at 6.8° can be attributed to the characteristic peak of carrageenan. Preliminary analysis suggests that when the proportion of carrageenan is too high, the system is prone to microphase separation, leading to local enrichment of carrageenan and the formation of aggregated regions with short-range ordered structures. In contrast, at lower carrageenan proportions, the higher starch content promotes uniform dispersion of the two phases through hydrogen bonding, inhibiting carrageenan aggregation, and thus this characteristic peak does not appear [29].

### 2.5. TGA of Hydroxypropyl Starch/Carrageenan Blends

**Figure 7:**
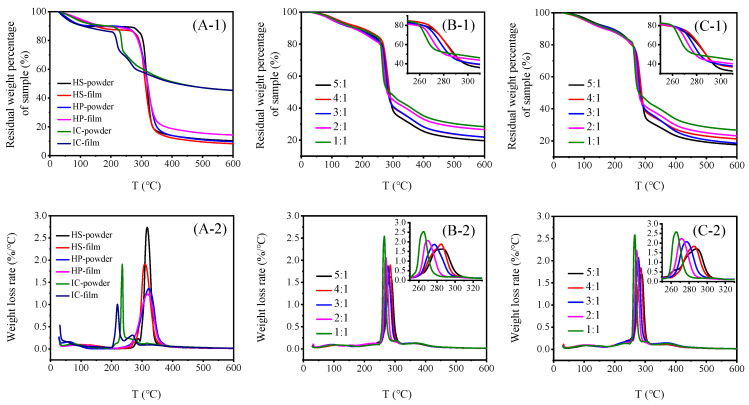
TGA (**A-1**–**C-1**) and DTG (**A-2**–**C-2**) curves for neat-component systems, hydroxypropyl amylose (HS)/carrageenan (IC) blends, and hydroxypropyl amylopectin (HP)/carrageenan (IC) blends.

Thermogravimetric analysis (TGA) is an analytical method used to determine the thermal properties of materials by measuring their mass change during heating. Figure 7 shows the TGA and derivative thermogravimetric (DTG) curves of hydroxypropyl starch, carrageenan, and their blends. The thermal decomposition process of the samples can be divided into two main stages: water evaporation and polymer thermal decomposition. The first stage occurs below 200 °C and corresponds to the gradual removal of free and bound water from the system. During this stage, the DTG curves changed gently, with relatively minor mass loss ranging from approximately 6.52% to 28.70%. Within this temperature range, hydroxypropyl starch exhibited higher thermal stability, with no significant differences in thermal behaviour between the samples. In contrast, carrageenan exhibited weaker thermal stability than hydroxypropyl starch.

The second stage mainly occurred in the range of 250–350 °C, corresponding to the thermal decomposition, chain scission, and gasification of the polymer backbone structure. The corresponding TGA parameters for the neat systems are summarized in Table 3. In this range, the maximum degradation temperature of the hydroxypropyl starch film was 321.69 °C, substantially higher than that of the carrageenan film (219.57 °C). This was attributed to the presence of sulfate groups in the carrageenan molecule, with higher degrees of sulfation leading to poorer thermal stability, whereas starch contains no sulfate groups, resulting in superior thermal stability and heat resistance of the starch film compared with the carrageenan film [30]. After high-temperature pyrolysis, the residual mass of the hydroxypropyl starch film at 600 °C was below 20%, whereas that of the carrageenan film was as high as 57.83%.

Table 4 shows the TGA parameters of the HSC and HPC blends. The blends exhibited a neat thermal degradation peak only, with peak temperatures falling between those of neat hydroxypropyl starch and neat carrageenan films. This indicates good molecular-level compatibility between hydroxypropyl starch and carrageenan. As the proportion of hydroxypropyl starch increased, the thermal decomposition rate of the blends decreased, the decomposition temperature range broadened and the maximum degradation temperature shifted significantly towards higher temperatures. Specifically, the degradation temperature of the 5:1 hydroxypropyl starch/carrageenan blend was approximately 18 °C higher than that of the 1:1 system, accompanied by a decrease in char residue. This suggests that the starch and carrageenan form a uniform, dense, physically cross-linked network via intermolecular hydrogen bonding. This effectively inhibits the movement and helix disintegration of the carrageenan molecular chains at high temperatures, thereby enhancing the blends’ overall thermal stability. Thus, the introduction of starch improves the compactness and thermal stability of the system. When comparing the two different hydroxypropyl starch/carrageenan blends, it was found that the molecular structural differences between amylose and amylopectin had no significant effect on the thermal properties of the films, with thermal stability being primarily influenced by molecular weight [31].

### 2.6. Texture Properties Analysis of Hydroxypropyl Starch/Carrageenan Blends

One-way analysis of variance (ANOVA) revealed extremely significant differences (*p* < 0.001) in all textural parameters among samples with different blending ratios, with degrees of freedom of (9, 20) for each group. The specific F-values and significance levels are presented in Table 5. Based on these results, Duncan’s multiple range test was conducted for multiple comparisons, and the results are shown in Table 6.

Texture profile analysis (TPA) is a widely used technique for quantifying the hardness and springiness of gel systems, as well as related mechanical attributes. Previous studies have demonstrated that various hydrocolloids influence starch gel hardness in different ways. For example, gellan gum and xanthan gum form tighter networks, increasing hardness, whereas guar gum weakens granule–granule interactions, resulting in a softer gel [32]. The textural parameters obtained for blends of hydroxypropyl amylose and hydroxypropyl amylopectin with carrageenan are summarized in Table 6. Neither the neat HS nor the neat HP system was capable of forming a self-supporting gel; however, all blends containing carrageenan at the same total solids concentration gelled readily. Since hydroxypropyl starch lacks gelling ability itself, the potential retrogradation phenomenon occurring after storage at 4 °C for 24 h made a very limited contribution to the hardness of the composite gel. Therefore, carrageenan was the key factor in determining the texture and properties of the blend.

In the present study, hardness rose markedly with increasing carrageenan content regardless of starch type. When the hydroxypropyl starch-to-carrageenan ratio was changed from 5:1 to 1:1, the hardness increased from 2.88 to 21.04 N for the HSC blends and from 3.99 to 23.13 N for the HPC blends—an increase of over 400% in both cases. This can be attributed to the progressively stronger intermolecular interactions and tighter molecular packing accompanying higher carrageenan loadings [33]. Chewiness and gumminess followed the same upward trend as hardness, peaking at the 1:1 ratio, as did adhesiveness.

Resilience and springiness, however, exhibited a rise-then-fall pattern, reaching their maxima at the 2:1 ratio for the HSC blend and the 3:1 ratio for the HPC blend. A plausible explanation is that moderate carrageenan additions promote elastic gel formation, boosting springiness and resilience, but beyond an optimal threshold the synergistic balance between the two hydrocolloids is disrupted, phase separation sets in, and the cohesive network structure is partially destroyed—leading to a decline in elastic recovery [34].

At equal blend ratios, the HPC blends generally exhibited superior texture. Taking the 1:1 formulation as an example, the amylopectin blend exhibited a hardness of 23.13 N, resilience of 0.70, and chewiness of 186.70 mJ, all surpassing the corresponding values for the amylose blend (21.04 N, 0.67, and 165.69 mJ), while also showing lower adhesiveness. During gelation, water acts as a medium to promote the movement and rearrangement of molecular chains. Under this condition, carrageenan, with its excellent gelling ability, dominates the properties of the blend, while starch plays a synergistic role. Owing to its branched structure, amylopectin swells during gelation and readily fills the gel skeleton formed by carrageenan, reinforcing the carrageenan network through space filling and skeleton stabilization. This significantly enhances the water-holding capacity of the blend, improves gel hardness and resilience, and reduces adhesiveness and other textural parameters. In contrast, amylose is less effective in promoting granule swelling and exhibits weaker water absorption and swelling capacity, resulting in inferior final textural properties compared with the HPC blends [35].

### 2.7. Mechanical Properties Analysis of Hydroxypropyl Starch/Carrageenan Blends

**Figure 8:**
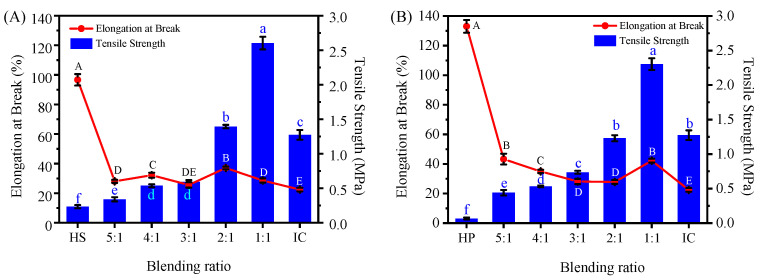
Tensile strength and elongation at break of hydroxypropyl amylose (HS)/carrageenan (IC) (**A**) and amylopectin (HP)/carrageenan (IC) (**B**) blends. Note: ANOVA showed extremely significant differences in tensile strength (F_6,14_ = 902.577) and elongation at break (F_6,14_ = 534.838) for the HSC system, as well as in tensile strength (F_6,14_ = 695.746) and elongation at break (F_6,14_ = 756.971) for the HPC system, with *p* < 0.001. Different uppercase or lowercase letters indicate statistically significant differences between groups (*p* < 0.05, *n* = 3).

Tensile strength and elongation at break are straightforward measures of a material’s mechanical toughness and rupture resistance. Figure 8 depicts the variation in these two parameters as a function of carrageenan content for the HSC and HPC blends. Neat hydroxypropyl starch films had low tensile strengths (0.23 MPa for HS and 0.07 MPa for HP) but high elongation at break values (96.64% and 133.02%, respectively). This behavior is intimately linked to molecular structure. During gelatinization, hydroxypropyl starch granules absorb water, swell, and disrupt their ordered chain arrangements, increasing the proportion of amorphous material. The resulting increase in chain flexibility and segmental mobility improves the extensibility of the starch film [36]. The film exhibited a tensile strength of 0.07 MPa and an elongation at break of 133.02%. Compared with the HP film, the HS film showed an approximately 228% higher tensile strength but a 27% lower elongation at break. This difference is attributed to the distinct molecular structures of the two starches. In the neat-component system, amylose formed a more ordered network, promoting stronger intermolecular interactions among starch molecules and resulting in tighter chain packing, which made the HS film harder and less extensible. In contrast, owing to the lack of sufficient long-range intermolecular interactions, the network structure formed by amylopectin exhibited lower strength, thus endowing the HP film with higher flexibility and lower tensile strength [37]. The neat carrageenan film, by contrast, showed a much higher tensile strength (approximately 1.27 MPa) but a low elongation at break of only 22.42%. This is consistent with the inherently brittle nature of carrageenan, whose strong interchain hydrogen bonding yields a rigid structure with limited extensibility once cross-linked with other polymers.

After blending hydroxypropyl starch with carrageenan, the tensile strength of the system gradually increased, while the elongation at break decreased. Taking the starch-to-carrageenan ratio of 1:1 as an example, the tensile strength of the blend was increased more than tenfold compared with the neat hydroxypropyl starch system, whereas the elongation at break decreased to approximately 30–40%. Although the elongation at break of the blend was substantially lower than that of the neat starch film, it was still higher than that of the neat carrageenan film (approximately 22.42%). This indicates that the addition of carrageenan significantly inhibits the flexibility and mobility of starch molecular chains, while the presence of starch simultaneously compensates for the excessive brittleness of the neat carrageenan film, allowing blends to achieve a better balance between strength and flexibility. This phenomenon originates from the dehydration process during film formation: as the system loses water support, the structure collapses to form a dense solid film layer, where the strength and compactness of the gel network directly determine the mechanical properties of the film. At this stage, carrageenan no longer acts as an elastic support but forms strong hydrogen bonds and electrostatic interactions with starch through its polar groups (hydroxyl and sulfate ester groups), enhancing the compactness and cohesive energy of the internal structure. Although this composite structure, with the carrageenan molecular chain network as the gel skeleton, significantly improves the tensile strength, the restricted movement of molecular chains leads to a decrease in elongation at break.

As the proportion of carrageenan increased, so did the tensile strength of the films. Under the same conditions, the HSC blends exhibited slightly higher strength than the HPC blends. This differs from the gel state, in which carrageenan dominates the system properties. After dehydration and film formation, the inherent rigidity and crystallizability of the molecular chains become dominant upon loss of the plasticizing effect of water. Consequently, the influence of starch type on the mechanical properties of the films becomes more pronounced. Specifically, the tensile strength of the composite films increased with amylose content; the higher the amylose content, the higher the tensile strength. This is because the linear structure of amylose readily forms crystalline regions. In contrast, the branched structure of amylopectin creates gaps between polymer chains. This weakens hydrogen bonding, consequently reducing tensile strength [38].

Owing to this structural difference, the HSC and HPC films exhibited different elongation at break trends when carrageenan was in excess. In the HSC films, the crystalline regions formed by amylose became excessively dense, severely restricting molecular chain slippage, causing the elongation at break to first increase and then decrease. In contrast, the branched structure of amylopectin in the HPC films maintained a certain degree of segmental mobility even with excess carrageenan, resulting in only a slight continuous increase in elongation at break without a decrease.

### 2.8. Surface Wettability Analysis of Hydroxypropyl Starch/Carrageenan Blends

**Figure 9:**
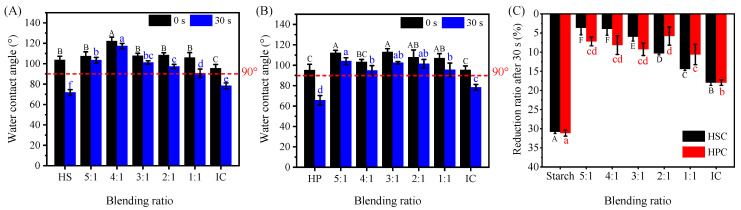
Initial contact angles of hydroxypropyl amylose (HS)/carrageenan (IC) (HSC, (**A**)) and hydroxypropyl amylopectin (HP)/carrageenan (IC) (HPC, (**B**)) blends, and the contact angle reduction ratio after 30 s (**C**). Note: ANOVA showed that the contact angle of the HSC blends varied significantly with time (0 s: F = 13.278, *p* < 0.001; 30 s: F = 92.881, *p* < 0.001; reduction ratio at 30 s: F_6,14_ = 238.216, *p* < 0.001). A similar significant difference was observed in the HPC blends (0 s: F = 7.569, *p* < 0.01; 30 s: F = 34.236, *p* < 0.001; reduction ratio at 30 s: F_6,14_ = 70.568, *p* < 0.001). Different uppercase or lowercase letters in the figure indicate statistically significant differences between groups (*p* < 0.05, *n* = 3).

The water contact angle is a commonly used indicator for evaluating surface hydrophobicity and wettability. A contact angle below 90° generally indicates a hydrophilic surface with good wettability, while a value above 90° signifies a hydrophobic surface with poor wettability. The surface wettability of the hydroxypropyl starch/carrageenan composite films can be preliminarily evaluated by measuring the contact angle, which provides a reference for assessing the material’s application performance. Figure 9 shows the initial contact angles for HSC (A) and HPC (B) blends, as well as the percentage decrease in contact angle within 30 s (C) at various carrageenan ratios. In the neat single-component system, the initial contact angle of HS was 103.67°, which is higher than that of HP (95.26°). Previous studies have shown that the ratio of amylose to amylopectin affects the hydrophobicity of starch-based blends. Starch-based films with higher amylose content possess higher crystallinity and a denser molecular packing structure, with smaller intermolecular gaps, thereby effectively inhibiting water molecule permeation and resulting in stronger hydrophobic characteristics.

After blending hydroxypropyl starch with carrageenan, the initial contact angles of all blends were higher than those of the neat components, exhibiting a trend of first increasing and then decreasing. Among them, the HSC blend achieved optimal hydrophobicity at a starch-to-carrageenan ratio of 4:1, with a contact angle of 122.18°, while the HPC blend reached optimal hydrophobicity at a 3:1 ratio, with a contact angle of 112.90°. These results suggest that introducing carrageenan can enhance the hydrophobicity of the blend. According to the Cassie-Baxter wetting model, droplets on a rough surface can exhibit an increased apparent contact angle due to the formation of a solid–liquid–gas three-phase interface. In this study, carrageenan may enhance the hydrophobic effect by altering the surface microstructure of the blend film and synergistically influencing the surface free energy [39].

Regardless of composition—whether neat starch, neat carrageenan, or a blend of the two—the contact angle consistently decreased within 30 s of droplet deposition (Figure 9C). This decrease stems from concurrent water evaporation and absorption into the film, increased moisture content in the matrix, and the formation of hydrogen bonds between polar groups on the surface and water molecules. These processes increase surface wetting. Notably, the magnitude of the 30 s decline was substantially smaller for the hydroxypropyl starch/carrageenan blends than for either the neat hydroxypropyl starch (30.00%) or the neat carrageenan (17.96%) system. These results suggest that the molecular interactions between carrageenan and starch alter the arrangement of surface molecules and the exposure of hydrophilic and hydrophobic functional groups. This effectively improves hydrophobicity and stabilizes the surface characteristics of the blend films.

### 2.9. Light Transmittance Analysis of Hydroxypropyl Starch/Carrageenan Blends

**Figure 10:**
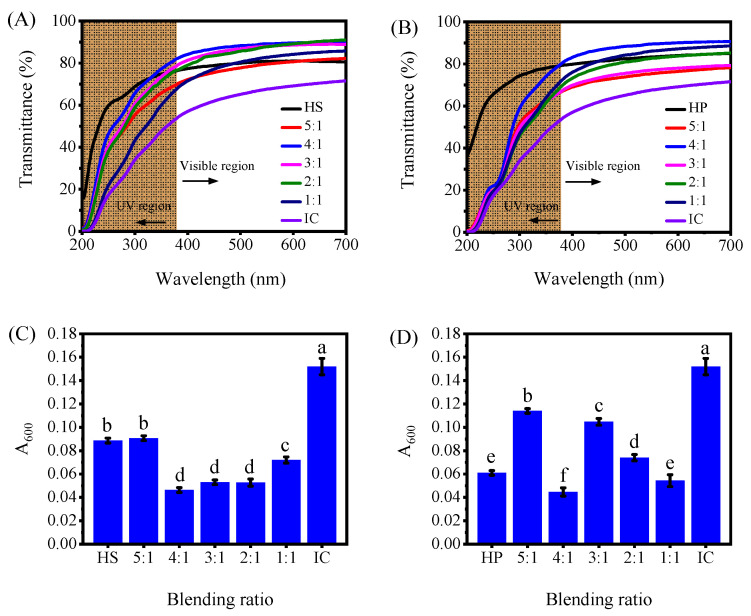
UV-Vis spectrophotometry of hydroxypropyl amylose (HS)/carrageenan (IC) blends (HSC, (**A**)) and hydroxypropyl amylopectin (HP)/carrageenan (IC) blends (HPC, (**B**)). The absorbance values at 600 nm, converted from transmittance, are presented in (HSC, (**C**)) and (HPC, (**D**)) for quantitative comparison. Note: ANOVA results showed that F_6,14_ = 341.923 (*p* < 0.001) for the HSC group and F_6,14_ = 282.764 (*p* < 0.001) for the HPC group. Different lowercase letters in the figure indicate statistically significant differences between groups (*p* < 0.05, *n* = 3).

Transmittance is a practical indicator of film homogeneity and component compatibility. The transmittance of the film relates to its internal structure, which forms during the drying process. Figure 10A,B shows the transmittance profiles of HSC and HPC blends with different carrageenan proportions scanned over the 200–700 nm range. Since the transmittance curves leveled off by 600 nm, the value at that wavelength was converted to absorbance and used for a more direct comparison between the two blends (Figure 10C,D). Looking first at the neat starch films, the neat HS film recorded a transmittance of 80.98%, which is noticeably lower than the 87.17% measured for the neat HP film. This is because the linear molecular chains of amylose readily form a dense structure with reduced void volume, thereby lowering light transmittance. In contrast, the branched structure of amylopectin hinders the orderly arrangement of molecular chains. This results in lower crystallinity and weaker internal scattering. Thus, light transmittance is higher.

The neat carrageenan film exhibited a low transmittance of only 69%. When blended with hydroxypropyl starch, the intermolecular interactions between the two components altered the microstructure and interfacial compatibility of the system, thereby affecting transmittance, with changes primarily governed by surface roughness and internal structure [40]. At an optimal carrageenan content, a synergistic effect occurred between the two components. Enhanced hydrogen bonding promoted an ordered arrangement of molecular chains, forming a denser and more uniform network structure. This improved film compactness and interfacial compatibility, optimized the microstructure, reduced phase separation, lowered surface roughness, and ultimately attenuated light scattering and increased transmittance. Conversely, excessive carrageenan competes with starch for water during solution blending, which inhibits complete dissolution and uniform dispersion of the starch. This leads to system heterogeneity, aggravated phase separation, and increased surface roughness. Consequently, light scattering increases and transmittance decreases [41]. As shown in Figure 10, at a starch-to-carrageenan mass ratio of 4:1, strong intermolecular interactions between the two components achieved the optimal synergistic effect, avoiding the water competition associated with excessive carrageenan while maximizing the reduction in light scattering through microstructural optimization. Consequently, the blend film achieved a transmittance of 90%, surpassing that of the neat starch system.

A material’s transparency directly affects consumers’ visual perception, so specific requirements should be determined according to the application scenario. The ultraviolet (UV) shielding performance of food packaging materials is also crucial because UV radiation readily induces lipid photooxidation and degrades photosensitive components. UV radiation also causes the photooxidative degradation of polymers, which deteriorates their mechanical properties. As Figure 10 shows, both hydroxypropyl starch and carrageenan are polysaccharides lacking conjugated structures, exhibiting no significant absorption in the visible light region. Although absorption bands are present in the 200–380 nm UV region, the overall absorption intensity is relatively weak. Among these, hydroxypropyl starch exhibits lower UV shielding capacity than carrageenan, while HP demonstrates slightly lower capacity than HS. When blended, the UV shielding performance of the blend falls between that of the individual components. HPC blend films demonstrate slightly better UV shielding efficiency than HSC blend films.

### 2.10. Multi-Index Comprehensive Comparison and Determination of the Optimal Ratio

**Figure 11:**
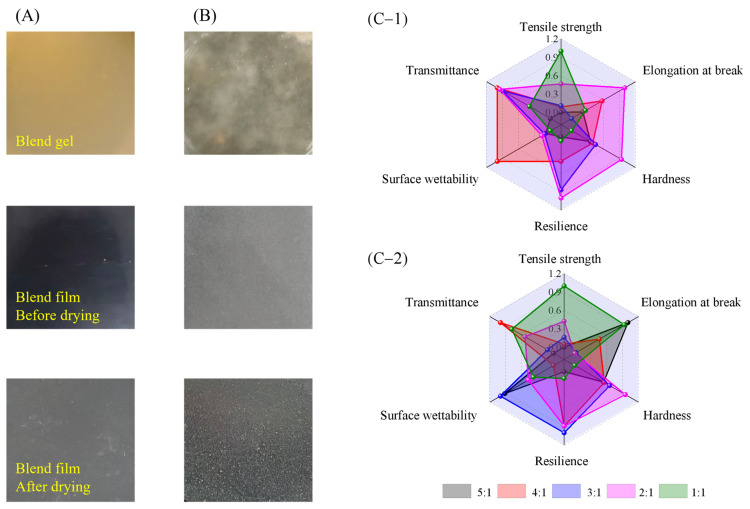
Apparent morphologies of gels, films before drying and after drying of hydroxypropyl starch/carrageenan blends at 2:1 (**A**) and 1:1 (**B**) ratios, and radar plot assessments of key performance indicators for hydroxypropyl amylose/carrageenan (HSC, (**C-1**)) and hydroxypropyl amylopectin/carrageenan (HPC, (**C-2**)) across various blend ratios.

Forming a uniform and continuous system is a prerequisite for endowing materials with their functional performance, whether in packaging materials, soft capsule shells, 3D printing, or other membrane/gel-related applications. A systematic evaluation was conducted on blends with different ratio combinations. The experimental results showed that the blend was uniform at low carrageenan ratios but deteriorated significantly when the ratio of carrageenan was excessive. Figure 11 shows the apparent morphologies of gels, films before drying, and films after drying at ratios of 2:1 and 1:1. With a 2:1 mass ratio of hydroxypropyl starch to carrageenan, the carrageenan content was relatively high. Although the color was somewhat dark, the blend was uniform, and the formed film had a smooth surface (Figure 11A). However, when the ratio increased to 1:1, significant phase separation occurred with carrageenan precipitation. This made it difficult to form a uniform gel, and the resulting film had a rough surface with no practical application value (Figure 11B). Therefore, the comprehensive evaluation prioritized the ratio range capable of forming a uniform and continuous blend, with “uniformity” as the primary screening indicator. Based on this, multi-dimensional indicators such as mechanical properties, hydrophobicity, and optical properties were considered. Key indicators were normalized to construct a radar chart. Based on the area of the radar chart, the ratio range of 2:1 to 4:1 was determined to be the optimal range for the two blends (Figure 11(C-1,C-2)). This evaluation logic ensures the fundamental formability of the material while avoiding sacrificing overall material usability in pursuit of individual properties.

## 3. Conclusions

This study compared the effects of hydroxypropyl amylose and hydroxypropyl amylopectin on starch/carrageenan blends. Rheological and structural characterizations revealed that, upon compounding hydroxypropyl starch with carrageenan, the blends’ rheological behavior transformed from weak viscoelasticity to a strong, elastic-dominated gel. FTIR analysis showed slight enhancement and narrowing of the -OH stretching peak. XRD patterns exhibited only a broad scattering peak. These results confirmed that the hydroxypropyl starch and carrageenan formed a homogeneous, dense, amorphous, three-dimensional network through hydrogen bonding. This network effectively improved the texture, mechanical strength, and stability of the blends. This lays the foundation for further investigation of the mechanism.

Combined with rheological, mechanical, and light transmittance tests, further analysis revealed that the structural advantages of amylose and amylopectin shifted with the system state, demonstrating obvious state-dependent effects. In the gel state, carrageenan’s gelling behavior was dominant. Amylopectin filled the gel skeleton via its high swelling power and branched structure. This resulted in the hydroxypropyl amylopectin/carrageenan blend having superior thermal resistance and texture properties. In the film state, the blend formed a dense solid film layer due to water loss. The effect of carrageenan weakened, while the structural difference in starch became the key optimizing factor. In the film state, the linear molecular chains of amylose readily form ordered crystalline domains, which impart superior properties to the blend films. Additionally, controlling the mass ratio of hydroxypropyl starch to carrageenan from 2:1 to 4:1 allowed both blends to avoid excessive cross-linking and decreased compatibility caused by excessive carrageenan. This achieved synergistic optimization of mechanical properties, hydrophobicity, and light transmittance. This study revealed the intrinsic relationship among starch molecular structure, system state, and macroscopic properties. This provides a theoretical basis for precisely regulating hydroxypropyl starch/carrageenan blends.

## 4. Materials and Methods

### 4.1. Materials and Instruments

High-amylopectin hydroxypropyl starch was purchased from Henan Hengrui Starch Technology Co., Ltd. (Luohe, China). High-amylose hydroxypropyl starch was obtained from Ingredion Incorporated (Westchester, IL, USA). Carrageenan was supplied by Shangfang Biotechnology Co., Ltd (Pinghu, China). Food-grade glycerol was purchased from Yantai Honghe Biotechnology Co., Ltd (Yantai, China). Purified water (Wahaha brand, Hangzhou Wahaha Group Co., Ltd., Hangzhou, China) was used throughout.

An AUW220D electronic balance (Shimadzu Corporation, Kyoto, Japan) was used for weighing. An HH.S21-6 thermostatic water bath was from Shanghai Boxun Medical Equipment Co., Ltd. (Shanghai, China). Fourier-transform infrared (FTIR) spectra were recorded on a Nicolet iS5 spectrometer (Thermo Fisher Scientific, Waltham, MA, USA). X-ray diffraction (XRD) patterns were collected with a D8 ADVANCE diffractometer (Bruker Corporation, Karlsruhe, Germany). Tensile tests were performed on an HDS-01 touch-screen universal testing machine (Jinan Hengxu Testing Machine Technology Co., Ltd., Jinan, China). Contact angle measurements were carried out with an SL200B goniometer (KINO Industrial Co., Ltd., New York, NY, USA). Rheological measurements were conducted on a MARS 60 rotational rheometer (Thermo Fisher Scientific, Karlsruhe, Germany). Transmittance was measured with a TU-1901 double-beam UV-Vis spectrophotometer (Beijing Purkinje General Instrument Co., Ltd., Beijing, China). Texture profile analysis (TPA) was performed on a TA-XTplus texture analyzer (Food Technology Corporation, Sterling, VA, USA). Viscosity was measured using an NDJ-T rotational viscometer (Shanghai Nirun Intelligent Technology Co., Ltd., Shanghai, China). Molecular weight was determined by an E2695 high-performance liquid chromatograph (Waters Technology Co., Ltd., Shanghai, China). Thermogravimetric analysis was performed on a TGA 8000 thermogravimetric analyzer (PerkinElmer Instruments Co., Ltd., Shelton, CT, USA).

### 4.2. Experimental Procedures

#### 4.2.1. Determination of Basic Physicochemical Properties of Hydroxypropyl Starch and Carrageenan

The pH, viscosity, hydroxypropyl content, and other basic indicators of hydroxypropyl starch (HPS) were determined according to the relevant methods specified in GB 29930-2013 [42]. Solubility was determined by drying the supernatant and calculating the percentage of leached solids relative to the initial dry weight of starch. Swelling power was determined as the weight ratio of the centrifuged swollen precipitate to the original dry starch mass, excluding soluble fractions.

The sulfate content, ash content, viscosity, pH, and other basic indicators of carrageenan were determined according to the relevant methods specified in GB 1886.169-2016 [43]. Gel strength was measured using a gelometer at a mass fraction of 1.5%.

The molecular weight distribution was determined by high-performance liquid chromatography (HPLC) equipped with a Model 2414 refractive index detector (Waters Corporation, Milford, MA, USA). An accurately weighed dried sample (2 mg) was dissolved in 2 mL of 0.2 mol/L NaCl solution, heated at 85 °C until fully dissolved, filtered through a 0.45 μm membrane, and then injected into the system. Using 0.2 mol/L NaCl as the mobile phase, the relative molecular weight distribution was obtained.

The preparation and characterization of the starch/carrageenan blends are described as follows [44].

#### 4.2.2. Preparation of Hydroxypropyl Starch/Carrageenan Blends

Hydroxypropyl starch and carrageenan were weighed at predetermined mass ratios (neat starch, 5:1, 4:1, 3:1, 2:1, and 1:1) to a total mass of 8.00 g and placed in a beaker, to which 2.80 g of glycerol and 72.00 g of water were added. The mixture was stirred thoroughly and then heated in a 100 °C water bath with continuous stirring for 60 min until complete dissolution. Water lost through evaporation during heating was recorded and replenished with ultrapure water, yielding a system with a solid content of approximately 13.04%. The resulting sol was transferred to an 85 °C water bath and held quiescently until all air bubbles were eliminated. A 50 mL aliquot was accurately transferred into a 100 mL beaker and stored at 4 °C for 24 h to allow stable gel formation; these gels were reserved for subsequent rheological and textural characterization. The remaining blended solution was accurately measured (45.00 g) and evenly poured into a polytetrafluoroethylene mold by solution casting, with the casting volume controlled to ensure uniform film thickness. The mold was placed in a vacuum drying oven at 50 °C with a vacuum degree controlled between 0.08–0.09 MPa. After drying for 6 h until the moisture content reached 15% ± 1.43%, the film was carefully peeled from the mold, yielding a film thickness of 0.60 mm ± 0.03 mm. The films were sealed in zip-lock bags and stored in a desiccator for hydrophobicity, light transmittance, and other property evaluations.

#### 4.2.3. Rheological Characterization

Strain sweep: A parallel-plate geometry (diameter 60 mm, gap 1.000 mm) was employed to identify the linear viscoelastic region (LVR) and to monitor the modulus evolution of each blend. Measurements were performed at 25 °C and a fixed frequency of 1 Hz over a strain range of 0.1–1000%.

Frequency sweep: Using the same geometry and gap setting, the frequency dependence of the storage and loss moduli was recorded at 25 °C and a fixed strain of 1% (within the LVR). Angular frequency (ω) was swept from 0.628 to 62.800 rad·s^−1^.

Temperature sweep: The temperature dependence of the moduli was evaluated at a frequency of 1 Hz and a strain of 1%, and the temperature was ramped from 25 °C to 90 °C.

For all rheological measurements, the sample surface was covered with soybean oil to prevent moisture loss during the test.

#### 4.2.4. Fourier-Transform Infrared Spectroscopy (FTIR)

Appropriate amounts of dried starch and carrageenan samples were gently ground with KBr powder in an agate mortar and pressed into pellets. FTIR spectra were acquired against a pure KBr background over the range of 400–4000 cm^−1^, accumulating 32 scans per sample at a resolution of 4 cm^−1^.

#### 4.2.5. X-Ray Diffraction (XRD)

Film specimens of suitable dimensions were mounted on the sample stage of the X-ray diffractometer. Scans were performed at 40 kV and 40 mA over a 2θ range of 4–50° with a step size of 0.02° and a counting time of 0.1 s per step.

#### 4.2.6. Thermogravimetric Analysis (TGA)

The thermal stability of the system was characterized using TGA. Approximately 4 mg of starch powder, carrageenan powder, and the trimmed composite film samples were weighed and uniformly placed in crucibles, respectively. Tests were performed from 30 to 580 °C at a heating rate of 10 °C·min^−1^ under a nitrogen atmosphere with a flow rate of 20 mL/min to eliminate interference from air.

#### 4.2.7. Texture Profile Analysis

Gels prepared in Section 4.2.2 were subjected to a two-cycle compression test using a flat-plate probe. Pre-test and post-test speeds were set at 60 mm·min^−1^, the test speed at 30 mm·min^−1^, the trigger force at 0.1 N, and the compression ratio at 40%.

#### 4.2.8. Mechanical Property Measurement

Film moisture content was equilibrated to 16.0% ± 0.4%, after which specimens were cut into 5 cm × 10 cm rectangles. Tensile strength and elongation at break were measured on the universal testing machine equipped with a tensile fixture at a pre-test and post-test speed of 50 mm·min^−1^ and a test speed of 10 mm·min^−1^.

#### 4.2.9. Surface Wettability Measurement

Static water contact angles were determined with the goniometer to assess surface hydrophilicity. Specimens were trimmed to 1 cm × 2 cm rectangles and affixed to glass slides. A 3.0 μL water droplet was rapidly deposited on the film surface at room temperature, and contact angles were recorded at 0 s and 30 s after droplet deposition.

#### 4.2.10. Light Transmittance Measurement

Baseline correction was performed with an empty quartz cuvette. Film specimens (1 cm × 2 cm) were cut and attached to the inner wall of a quartz cuvette. Transmittance spectra were acquired on the UV-Vis spectrophotometer over 200–700 nm at 1 nm intervals. For a more intuitive quantitative comparison, the transmittance at 600 nm (T) was converted to absorbance (A) using the definition of absorbance.

#### 4.2.11. Statistical Treatment

For qualitative and spectral characterizations such as Fourier transform infrared spectroscopy (FTIR), thermogravimetric analysis (TGA), X-ray diffraction (XRD) and rheological properties, only representative spectra or curves are displayed, without calculating the mean and standard deviation. All quantitative indicators, including mechanical properties, texture properties, hydrophobicity and light transmittance, are expressed as mean ± standard deviation (Mean ± SD). Each data point represents the result of three independent experiments with three replicates per experiment. Error bars in the figures represent the standard deviation. Statistical analysis and table plotting were performed using IBM SPSS Statistics 26 (IBM Corp., Armonk, NY, USA), and differences between groups were tested by Duncan’s multiple range test at a significance level of *p* < 0.05, and figures were plotted using Origin 2021 (OriginLab Corp., Northampton, MA, USA).

## Figures and Tables

**Table 1:** Basic physicochemical properties of hydroxypropyl amylose (HS) and hydroxypropyl amylopectin (HP) measured in triplicate (*n* = 3).

Type	Amylose Content	Viscosity (mPa·s)	Hydroxypropyl Content (%)	Moisture (%)	pH	Solubility (%)		Swelling (g/g)	
						25 °C	75 °C	25 °C	75 °C
HS	>70%	20.10 ± 1.20	4.33 ± 0.05	11.76 ± 0.12	6.2	1.05 ± 0.07	93.11 ± 1.44	1.48 ± 0.32	20.39 ± 0.21
HP	<5%	396.00 ± 4.80	4.30 ± 0.02	10.98 ± 0.42	6.2	2.40 ± 0.12	75.90 ± 3.23	2.96 ± 0.22	61.56 ± 0.37

**Table 2:** Basic physicochemical properties of carrageenan (*n* = 3).

Type	Viscosity (mPa·s)	Sulfate Ester Content (%)	Ash Content (%)	Gel Strength (g)	Moisture (%)	pH
ι–carrageenan	64.40 ± 1.20	28.30 ± 0.31	20.98 ± 0.40	9.00 ± 0.00	9.68 ± 0.17	7.5

**Table 3:** TGA parameters of hydroxypropyl amylose (HS), hydroxypropyl amylopectin (HP), and ι-carrageenan (IC) in powder and film forms.

Sample	Form	Onset Temperature (°C)	Peak Temperature (°C)	Residual Mass at 600 °C (%)
HS	powder	255.83	318.02	15.54
	film	247.37	311.05	12.95
HP	powder	205.28	321.69	13.35
	film	227.58	317.43	18.55
IC	powder	190.04	234.64	60.66
	film	183.94	219.57	57.83

**Table 4:** TGA parameters of hydroxypropyl amylose/carrageenan (HSC) and hydroxypropyl amylopectin/carrageenan (HPC) blends.

Sample	Ratio	Onset Temperature (°C)	Peak Temperature (°C)	Residual Mass at 600 °C (%)
HSC	5:1	225.50	283.43	33.74
	4:1	207.26	281.32	37.46
	3:1	239.03	277.57	38.28
	2:1	236.13	271.16	42.22
	1:1	177.66	265.10	48.50
HPC	5:1	182.69	284.9	30.67
	4:1	175.43	283.19	34.97
	3:1	166.18	277.37	36.28
	2:1	205.24	272.48	39.38
	1:1	235.13	266.36	45.85

**Table 5:** ANOVA of texture parameters for hydroxypropyl starch/carrageenan blends at various ratios (*n* = 3).

Parameter	Hardness (N)	Resilience	Adhesiveness (mJ)	Cohesiveness	Springiness	Gumminess (mJ)	Chewiness (mJ)
F value	3363.740	100.376	2438.164	17.296	192.301	908.013	678.164
*p* value	<0.001	<0.001	<0.001	<0.001	<0.001	<0.001	<0.001

Note: Degrees of freedom df = 9, 20 (where df_1_ = 9 is the between-group degrees of freedom, and df_2_ = 20 is the within-group degrees of freedom); *p* < 0.001 indicates an extremely significant difference.

**Table 6:** Texture parameters of hydroxypropyl amylose/carrageenan (HSC) and hydroxypropyl amylopectin/carrageenan (HPC) blends.

Starch Type	Ratio	Hardness (N)	Resilience	Adhesiveness (mJ)	Cohesiveness	Springiness	Gumminess (mJ)	Chewiness (mJ)
HSC	1:1	21.04 ± 0.34 ^b^	0.67 ± 0.01 ^f^	1.56 ± 0.05 ^a^	0.94 ± 0.03 ^ab^	8.38 ± 0.10 ^c^	19.76 ± 0.84 ^b^	165.69 ± 9.06 ^a^
	2:1	10.68 ± 0.12 ^c^	0.81 ± 0.01 ^b^	0.47 ± 0.01 ^d^	0.86 ± 0.04 ^e^	8.79 ± 0.02 ^b^	9.24 ± 0.21 ^c^	81.40 ± 2.05 ^c^
	3:1	3.94 ± 0.06 ^e^	0.79 ± 0.01 ^c^	0.65 ± 0.00 ^c^	0.91 ± 0.01 ^bc^	8.70 ± 0.08 ^b^	3.61 ± 0.10 ^e^	31.39 ± 0.77 ^ef^
	4:1	3.37 ± 0.13 ^f^	0.72 ± 0.02 ^d^	0.47 ± 0.01 ^d^	0.94 ± 0.01 ^ab^	7.05 ± 0.04 ^e^	3.16 ± 0.16 ^e^	22.27 ± 1.00 ^gh^
	5:1	2.88 ± 0.07 ^j^	0.66 ± 0.01 ^f^	0.24 ± 0.00 ^e^	0.81 ± 0.01 ^f^	6.65 ± 0.07 ^f^	2.36 ± 0.09 ^f^	15.70 ± 0.73 ^h^
HPC	1:1	23.13 ± 0.55 ^a^	0.70 ± 0.02 ^e^	1.40 ± 0.02 ^b^	0.95 ± 0.02 ^a^	8.47 ± 0.02 ^c^	22.04 ± 0.81 ^a^	186.70 ± 7.11 ^b^
	2:1	10.66 ± 0.06 ^c^	0.79 ± 0.01 ^c^	0.45 ± 0.01 ^d^	0.86 ± 0.02 ^e^	8.79 ± 0.08 ^b^	9.30 ± 0.10 ^c^	81.77 ± 1.61 ^c^
	3:1	5.48 ± 0.03 ^d^	0.87 ± 0.02 ^a^	0.15 ± 0.00 ^f^	0.87 ± 0.01 ^de^	9.48 ± 0.02 ^a^	4.77 ± 0.05 ^d^	45.25 ± 0.53 ^d^
	4:1	4.14 ± 0.12 ^e^	0.86 ± 0.02 ^a^	0.14 ± 0.00 ^f^	0.90 ± 0.01 ^cd^	8.90 ± 0.08 ^b^	3.70 ± 0.13 ^e^	32.97 ± 1.44 ^e^
	5:1	3.99 ± 0.10 ^e^	0.78 ± 0.01 ^c^	0.16 ± 0.00 ^f^	0.84 ± 0.03 ^ef^	7.61 ± 0.29 ^d^	3.36 ± 0.01 ^e^	25.54 ± 0.99 ^g^

Note: Different lowercase superscript letters in the same column indicate statistically significant differences between group means (*p* < 0.05, *n* = 3).

## Data Availability

The data presented in this study are available on request from the corresponding author.
